# A 17-Gene Expression Signature for Early Identification of Poor Prognosis in Clear Cell Renal Cell Carcinoma

**DOI:** 10.3390/cancers14010178

**Published:** 2021-12-30

**Authors:** Maria Bassanelli, Marina Borro, Michela Roberto, Diana Giannarelli, Silvana Giacinti, Simona Di Martino, Anna Ceribelli, Andrea Russo, Annamaria Aschelter, Stefania Scarpino, Andrea Montori, Edoardo Pescarmona, Silverio Tomao, Maurizio Simmaco, Francesco Cognetti, Michele Milella, Paolo Marchetti

**Affiliations:** 1 Department of Medical and Surgical Sciences and Translational Medicine, Faculty of Medicine and Psychology, Sapienza University of Rome, 00100 Rome, Italy; maria.bassanelli@yahoo.it; 2 (DIMA) Department of Neurosciences, Mental Health and Sensory Organs, Sapienza University of Rome, 00187 Rome, Italy; marina.borro@uniroma1.it; 3 Department of Radiological, Oncological and Anatomo-Pathological Sciences, Medical Oncology Unit, Umberto I University Hospital, Sapienza University of Rome, 00185 Rome, Italy; silverio.tomao@uniroma1.it; 4 Clinical Trial Center, Biostatistics and Bioinformatics, IRCCS Regina Elena National Cancer Institute, Via Elio Chianesi, 53, 00144 Rome, Italy; diana.giannarelli@ifo.gov.it; 5 Department of Oncology, Sant’Andrea Hospital, 00187 Rome, Italy; silvana.giacinti@aslfrosinone.it (S.G.); Annamaria.aschelter@ospedalesantandrea.it (A.A.); 6 Department of Pathology, IRCCS Regina Elena National Cancer Institute, Via Elio Chianesi, 53, 00144 Rome, Italy; simona.dimartino@ifo.gov.it (S.D.M.); andrea.russo@ifo.gov.it (A.R.); edoardo.pescarmona@ifo.gov.it (E.P.); 7 Department of Oncology, San Camillo de Lellis Hospital, Viale Kennedy, 12100 Rieti, Italy; a.ceribelli@asl.rieti.it; 8 Department of Clinical and Molecular Medicine, Pathology Unit, St. Andrea University Hospital, University of Rome La Sapienza, 00187 Rome, Italy; stefania.scarpino@uniroma1.it (S.S.); andrea.montori@uniroma1.it (A.M.); 9 Department of Neurosciences, Mental Health and Sensory Organs (NESMOS), Advanced Molecular Diagnostic Unit (Dima), Sapienza University, Sant’Andrea Hospital, 00187 Rome, Italy; maurizio.simmaco@uniroma1.it; 10 Medical Oncology 1, IRCCS Regina Elena National Cancer Institute, Via Elio Chianesi, 53, 00144 Rome, Italy; francesco.cognetti@ifo.gov.it; 11 Division of Oncology, Integrated University Hospital of Verona, Via S. Francesco 22, 37129 Verona, Italy; michele.milella@univr.it; 12 Department of Clinical and Molecular Medicine, Oncology Unit, Sant’ Andrea Hospital, Sapienza University of Rome, 00187 Rome, Italy; paolo.marchetti@uniroma1.it

**Keywords:** clear cell renal cancer cell (ccRCC), prognosis, Next-Generation Sequencing (NGS), Next-Generation expression analysis (NanoString), recurrence, biomarker

## Abstract

**Simple Summary:**

Our analysis of a 17-gene expression signature resulted in being significantly different among patients with clear cell renal cancer cell (ccRCC) who reported a recurrence-free survival (RFS) >5 years and patients with a RFS < 1 year. This Genomic Signatures could be useful to better identify good prognosis (with favorable genomic signature) against poor prognosis (with unfavorable genomic signature) ccRCC. Accordingly, both follow-up and treatment could be profoundly personalized for patients with neodiagnosis of ccRCC in the near future.

**Abstract:**

The Identification of reliable Biomarkers able to predict the outcome after nephrectomy of patients with clear cell renal cell carcinoma (ccRCC) is an unmet need. The gene expression analysis in tumor tissues represents a promising tool for better stratification of ccRCC subtypes and patients’ evaluation. Methods: In our study we retrospectively analyzed using Next-Generation expression analysis (NanoString), the expression of a gene panel in tumor tissue from 46 consecutive patients treated with nephrectomy for non-metastatic ccRCC at two Italian Oncological Centres. Significant differences in expression levels of selected genes was sought. Additionally, we performed a univariate and a multivariate analysis on overall survival according to Cox regression model. Results: A 17-gene expression signature of patients with a recurrence-free survival (RFS) < 1 year (unfavorable genomic signature (UGS)) and of patients with a RFS > 5 years (favorable genomic signature (FGS)) was identified and resulted in being significantly correlated with overall survival of the patients included in this analysis (HR 51.37, *p* < 0.0001). Conclusions: The identified Genomic Signatures may serve as potential biomarkers for prognosis prediction of non-metastatic RCC and could drive both follow-up and treatment personalization in RCC management.

## 1. Introduction

Renal Cell Carcinoma (RCC) accounts for approximately 3% of all malignancies [1]. In Italy, it causes more than 3.717 deaths/year and the incidence of new cases is estimated at approximately 12,600 new cases/year [2]. The most frequent (70–85%) histologic subtype is the clear cell renal cell carcinoma (ccRCC), a highly vascular tumor arising from the proximal tubules of [3]. The treatment of choice for early-stage disease is radical or partial nephrectomy; however, about 50% of subjects with clinically localized disease will eventually relapse, and two-thirds of them will recur within the first year [4,5,6]. Further, effective adjuvant therapies have not been established yet, since VEGF TKIs have failed in high-risk (pT3, pT4, node-positive), resected RCC, and the ASSURE [7,8], S-TRAC [9,10] and PROTECT trials [11]. Using Sorafenib, Sunitinib, and Pazopanib in an adjuvant setting did not reveal an improvement in DFS and OS. The double-blind, phase 3 trial, KEYNOTE-564, showed that patients with clear-cell renal-cell carcinoma who were at high risk for recurrence after nephrectomy, with or without metastasectomy, who received adjuvant pembrolizumab therapy had significantly longer disease-free survival than placebo (disease- free survival at 24 months, 77.3% vs. 68.1%; hazard ratio for recurrence or death, 0.68; 95% confidence interval [CI], 0.53 to 0.87; *p* = 0.002 [two-sided]) [12]. According to this data on the role of pembrolizumab as adjuvant treatment, the identification of a prognostic gene signature could be very helpful for better selection of those patients at higher risk of recurrence who may benefit from adjuvant treatment.

Presently, two clinical models can be used to evaluate the risk of ccRCC progression: the Mayo Clinic Stage, Size, Grade, and Necrosis (SSIGN) [13] score and the University of California Los Angeles Integrated Staging System (UISS) [14]. The SSIGN system analyzes histological features as TNM tumor stage (*p* < 0.001), size ≥ 5 cm (*p* < 0.001), nuclear grade (*p* < 0.001), and tumor necrosis (*p* < 0.001) and assigns a risk score from 3 to 10, the higher the score, the shorter the median survival.

The UISS model evaluates histological parameters like TNM stage, Fuhrman grade, and Eastern Cooperative Oncology Group performance status and stratifies patients with localized RCC in low, intermediate, and poor risk groups with 5-year survival rates of 92%, 67%, and 44%, respectively.

The ccRCC is characterized by high molecular heterogeneity, as pointed out by the number of involved driver genes. Even if the most prevalent loss-of-function mutation in ccRCC affects the von Hippel–Lindau (VHL, 44–90% of cases) [15], gene mutations have also been identified in PBRM1 (32–41%), BAP1 (6–15%), SETD2 (3–11%), TP53, (5%), KDM5C (3–5%), PIK3CA (3%), TSC1 (3%), ARID1A (2%), and CDKN2A (2%). Dissection of the molecular heterogeneity characterizing the tumor tissues is, thus, of paramount importance to explain the landscape of clinical manifestations, progression risk, and differential response to pharmacological therapies. In particular, gene expression analysis in tumor tissues represents a promising tool for better stratification of ccRCC subtypes and patients’ evaluation. Previous studies reported ccRCC gene expression signatures associated with prognosis, as the ClearCode34 [15,16,17], or associated with recurrence risk and therapy response [18,19]. The molecular markers identified by different studies differ according to the patient selection criteria (tumor type, stage, grade, therapies) and to the selected outcome (survival, progression, recurrence). Therefore, presently there are no validated gene expression prognostic biomarkers applicable in all ccRCC setting. Further, to enhance clinical adoption of sophisticated molecular diagnostic panels, such as gene expression analysis, a better performance compared to histological/clinical evaluation should be demonstrated.

In recent years, a novel, medium-throughput (up to 800 genes analyzed simultaneously) technology for gene expression analysis, that is, the Nanostring nCounter system [20], allows implementing gene signatures in clinical practice [21]. We developed a Nanostring 195-plexed gene expression panel for ccRCC evaluation, including probes for (1) the most relevant (to our knowledge) prognostic gene signatures reported in literature, (2) genes frequently mutated in ccRCC, and (3) genes reported as differentially expressed in ccRCC compared to normal tissue (see Materials and Methods). In this retrospective study, we employed this analytical panel to compare gene expression between non-metastatic ccRCC patients who had a recurrence-free survival (RFS) of < 1 year and non-metastatic ccRCC patients who had an RFS of >5 year.

## 2. Materials and Methods

### 2.1. Patients

This was an observational, case-control, retrospective, multicenter study, including 46 adult patients (age ≥ 18) referred to the Sant’Andrea Hospital “Sapienza” University of Rome and “Regina Elena” National Cancer Institute of Rome in the period 2012–2018. Patients were consecutively enrolled, according to the stage at the time of diagnosis (stage I–III ccRCC) and treated with nephrectomy (defined as “partial” or “radical”) with no previous systemic therapy. After that surgery, no adjuvant treatment was done. Patients were excluded if biopsies’ samples were derived from metastases. Follow-up of patients was performed according to the standard of care at the participating institutions. The SSIGN scoring system (1997 TNM stage, tumor size ≥ 5 cm nuclear grade, and histological tumor necrosis) was used to define the aggressiveness of ccRCC.

Patients were categorized according to the RFS, defined as the time from the date of surgery to the date of recurrence or last follow-up. The group with unfavorable genomic signature (UGS, N = 22) had an RFS of <1 year and the favorable genomic signature (FGS, N = 24) had an RFS of >5 years. Data collected from medical records and pathology reports included Karnofsky performance status (PS), diameters of the primary tumors, Fuhrman grade, lymph node involvement, tumor necrosis, sarcomatoid component, surgery (cytoreductive, partial or radical nephrectomy), tumor stage, date of radiographic or clinical progression, and date of death or last follow-up. Overall survival (OS) was calculated from the time of surgery to the date of death for any cause or last follow-up.

Ethical approval for this study was obtained by the local committees (Prot. n. 107 SA_2017 del 19.04.2017; RIF. CE: 4407) and patients provided written, informed consent.

### 2.2. Gene Expression Panel Selection

The full list of selected genes, with probes’ sequences and isoform coverage, is reported in the Supplementary Table S1. The gene expression panel was designed to include genes previously identified as prognostics’ factors [16,19], gene-defining molecular subtypes according to Beuselinck et al. [16], genes identified as differentially expressed between BAP1- and PBRM1-mutant tumors [22], genes involved in energy metabolism and in ccRCC [23], and genes from the ccRCC dataset from The Cancer Genome Atlas (TCGA) [24]. Nine housekeeping genes were also included.

### 2.3. RNA Extraction and Nanostring Analysis

Total RNA was extracted using the High Pure FFPET RNA Isolation Kit (Roche, Basilea, Switzerland), according to the manufacturer’s protocol, and quantified using the NanoDrop2000 spectrophotometer (Thermo Fisher Scientific, Waltham, DE, USA). After evaluation of RNA size and integrity using the 2100 Bioanalyzer (Agilent Technologies, Santa Clara, CA, USA), samples were stored at −80 °C until analysis. RNA samples were excluded from the analyses if they had concentrations <25 ng/μL or a RNA Integrity Number value < 6.5.

Nanostring analysis was performed according to the manufacturer’s instructions. Briefly, 5 μL of each sample were mixed with 8 μL of the hybridization cocktail containing the reporter code set. Then, 2 μL of the capture code set were added. Hybridization was performed in a 65 °C thermocycler (Veriti Thermal Cycler, Applied Biosystems, Foster City, CA, USA) for 18 h. The samples were then loaded onto the Nanostring cartridge using the automated Nanostring prep station, and the cartridge was scanned with the Nanostring Digital Analyzer to obtain probes’ counts.

### 2.4. Statistics

#### 2.4.1. Sample Size Calculation

In order to determine sample size, we focused on the False Discovery Rate (FDR). This rate depends strongly on the formula (1-π)/α where α is the type I error and π is the proportion of the genes that are not differentially expressed between the two compared groups. The value of (1-π) is typically in the range of 0.005 to 0.05, but in our study we expected a higher rate because of the candidate gene approach we used and because the two groups are highly different in prognostic terms (patients with RFS of <1 year and patients with RFS of >5 years). The ssize.fdr package version 1.2 (R version 2.14.2) was used. Assuming an effect size of 0.80, a power of 80%, and a FDR = 0.05, a range of different sample sizes according to π were investigated. On the basis of this calculation and of availability of retrospective information and practical consideration, a total of 46 patients were included in the study (24 patients with RFS of < 1 year and 22 with RFS of > 5 years); this sample size allowed us to assess a π = 0.90. Differences between patients’ characteristics in the two groups were assessed by chi-square test when related to categorical variables and by Mann–Whitney test when considering quantitative items. Cox proportional hazard model was used to estimate hazard ratios and their 95% confidence intervals.

The primary end point was the identification of an UGS associated with worse prognosis (RFS < 1 year) and of a FGS associated with a better prognosis (RFS > 5 years). The gene expression signature associated with poor prognosis was assessed by tumor characteristics, including the SSIGN (Stage, Size, Grade, and Necrosis) score. The SSIGN score, composed of four clinical assessment measures (7th version TNM classification system, tumor size ≥ 5 cm, nuclear grade, and histological tumor necrosis), was used to define aggressiveness of ccRCC.

#### 2.4.2. Nanostring Data Analysis

The raw data file from the Nanostring Digital Analyzer was analyzed by the Nanostring nCounter nSolver™ 4.0 using the Nanostring Advanced Analysis Module 2.0 plugin. The Advanced Analysis Module 2.0 software uses open-source R programs for quality control (QC), normalization, and differential expression (DE) analysis. The analysis panel included six positive control probes with known expected counts’ number and eight negative control probes to test for analytical quality of each experimental run. Thus, technical normalization was performed using nSolver™ 4.0, according to the Nanostring Gene Expression Data Analysis Guidelines (Nanostring MAN-C0011-04), before running the Advanced Analysis Module 2.0. Biological normalization was then achieved by selecting the best reference probes among the nine housekeeping genes included in the panel. These reference genes (ACTB, CLTC, VDAC2, PGK1, B2M) were selected using the geNorm function of the Advanced Analysis Module, which ranks housekeeping genes according to the minimum expression variance among samples. DE analysis between the PP group and the GP group was performed using batch and cartridge IDs factored as confounding factors.

### 2.5. Study Design

This study design considered two groups of patients who were very different in prognostic terms. In the first step, the gene finding and the standard t statistic were used for the ranking; the t statistic is calculated as the difference in the class-specific means of log expression divided by an estimate of the standard error of the difference. In order to control the ‘‘false discovery rate’’ (FDR), a stringent threshold of significance was used (*p* = 0.01). Considering a total of 195 genes to be investigated, the expected number of false positives was about two genes; the FDR was given by the ratio between 2 and the number of genes for which the univariate significance level was less than 0.01.

The second step consisted of the development of a predictive classifier. A class predictor, or predictive classifier, is a computable function that can be used to predict a class from an expression profile. As suggested by Simon, in developing this predictive classifier, the principal aim was the predictive accuracy, sensitivity, specificity, and positive and negative predictive values and not the goodness of fit to the data or the statistical significance of regression coefficients.

The third step was the validation of this classifier, and a cross-validation method was implemented based on the leave-one-out procedure. The cross-validated prediction error is an estimate of the prediction error associated with the application of the algorithm for model building to the entire dataset.

At the end of the entire process, the classifier was completely specified (including cutoffs) to allow validation in external independent sets.

## 3. Results

According to RFS, patients were divided in two groups: 24 patients in the group with a RFS of >5 years versus 22 patients with an RFS of < 1 year. The two study groups were well balanced in terms of sex, tumor grade, necrosis, and sarcomatoid component representation in the histology, whereas the median age, disease stage, Karnofsky PS, and surgery done were significantly different between the two groups (Table 1). Differential analysis of gene expression between the RFS ≤ 1 year and RFS > 5 years groups identified 17 genes that maintained significantly different expression levels after Bonferroni correction (Table 2). This molecular signature was entirely down regulated in the RFS ≤ 1-year group compared to the RFS > 5-years group and was thus named as UGS.

**Figure 1:**
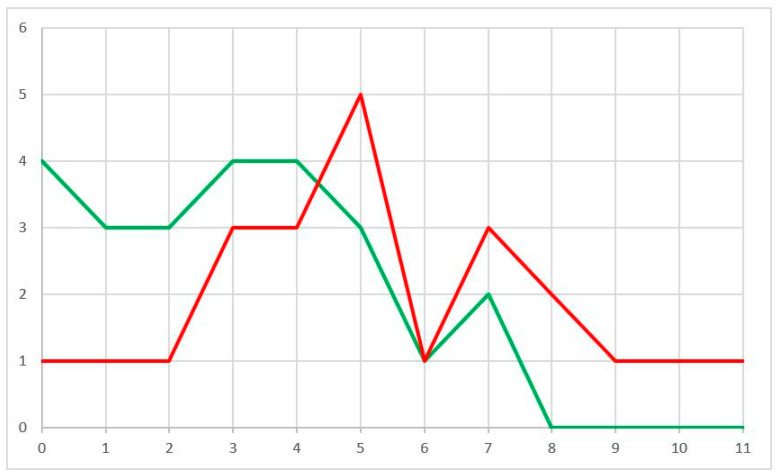
SSIGN score distribution in the two groups (RFS > 5 years (green) and RFS < 1 year (red)). X = SSIGN; Y = n pts.

Furthermore, we compared our risk assessment tool with existing clinical nomogram to predict death from ccRCC. SSIGN score distribution in two prognostic groups was very heterogeneous (Figure 1), suggesting an independent contribution of this signature to prognosis.

As expected, those patients who reported a RFS from the time of surgery of >5 years and a FGS had a longer OS than patients who reported a RFS of < 1 years and UGS. The univariate analysis on OS according to Cox regression model showed an association between Karnofsky Performance Status (*p* < 0.0001), tumor stage (*p* = 0.03), and SSIGN score (*p* = 0.003) and signature (*p* < 0.0001); no association was found for gender (*p* = 0.90), age (*p* = 0.11), grade (*p* = 0.78), necrosis (*p* = 0.75), and type of surgery (*p* = 0.08). (Table 3) However, at the multivariate analysis, when significant associations were tested simultaneously, and not considering signature, only Karnofsky (*p* < 0.0001) and SSIGN (*p* = 0.006) maintained their significance. When introducing signature, this was the only significant parameter (HR 51.34 (6.75–390.96); *p* < 0.0001)) (Table 3).

## 4. Discussion

We identified a 17-gene expression signature to predict the outcome of ccRCC patients. The primary endpoint was the identification of an unfavorable genomic signature associated with worse prognosis (RFS of <1 year) and of a favorable genomic signature associated with a better prognosis (RFS of >5 years). The gene expression signature associated with poor prognosis was assessed by tumor characteristics, including the SSIGN score. In this analysis, we distinguished two different subtypes of ccRCC characterized by different outcomes. Overexpression of a gene involved in infection/inflammation, PI3K-Akt signaling, HIF-1 signaling, and pentose phosphate pathway, was associated with an increased risk of recurrence.

The TCGA data showed the correlation between disease aggressiveness and metabolic shift that involved increased dependence on pentose phosphate shunt, downregulation of AMP-activated protein kinase (AMPK), and the Krebs cycle and increased glutamine transport and fatty acid production [25]. Additionally, Zhang Q. and colleagues showed the association between overexpression of glucose 6-phosphate dehydrogenase (G6PD) with poor prognosis [26].

In our study, patients with UGS were characterized by overexpression of POLD4. Interestingly, the role of POLD4 is a gene involved in mismatch repair, base excision repair, DNA replication, homologous recombination, and nucleotide excision repair. POLD4 downregulation activates checkpoint proteins, induces G1-S arrest, and delays the cell cycle from S to G2. POLD4 reduction induces also modest genomic instability, while allowing cells to grow until DNA damage reaches an intolerant level [27,28]. Its role in ccRCC is unclear.

USG was characterized by a high expression of interlekin-6 (IL-6), a gene involved in infection/inflammation that seems to play a pro-tumorigenic role, contributing to proliferation and invasion of the tumor cell. Wang et al. [29] showed the correlation between a high level of IL-6 and poor survival in RCC patients. Multigenic assay by Rini included IL-6 in inflammatory pathway [19]. Some studies showed the association between a high level of IL-6 and tyrosine kinase inhibitors’ resistance. IL6 is shown to be closely related to hypoxia inducible factor 1-α (HIF-1α) as well as increased VEGF activity. IL-6 binds IL-6 Receptor and results in activation of the JAK/STAT3 signaling pathway, leading to the transcription of STAT3 target genes, i.e., VEGF or SOCS3. SOCS3 suppresses STAT1 activation. STAT3 and NF-κB interact at multiple levels and promote inflammation, increasing tumor cell proliferation and survival as well as tumor angiogenesis and metastasis [30,31,32,33]. Motzer RJ presented at the 2020 ASCO annual virtual meeting the biomarker analyses from the phase 3 ChekMate 214 trial of nivolumab plus ipilimumab vs. sunitinib in clear-cell advanced renal cell carcinoma [34,35]. The angiogenesis gene signature was associated with ORR for sunitinib (high score) and Nivolumab + Ipilimumab (low score). Prolonged PFS with Nivolumab + Ipilimumab was associated with higher expression of Hallmark inflammatory response and Hallmark epithelial–mesenchymal transition gene sets. These results suggest the knowledge that our signature could also play an important role in the therapeutic choice (immunotherapy vs. TKI vs. TKI +immunotherapy).

As shown also with a ClearCode 34-based model, these genomic models were better predictors for recurrence than the SSIG Model [16,36].

Adjuvant treatment with the VEGF receptor tyrosine kinase inhibitors showed no survival benefit in resected, high-risk ccRCC. Therefore, our genomic signature can also explain the hypothesis that the biology of cancer recurrence might be independent of angiogenesis in this setting [37,38,39]: Micrometastases in the adjuvant setting do not require the support of tumor angiogenesis. Therefore, a VEGFR TKI would not eradicate micrometastases. Currently, the role of immune checkpoint inhibitors in an adjuvant setting shows promising results, but are still under investigation in ongoing trials.

Our 17-gene expression signature was significantly associated with RFS of < 1 year and showed greater superiority in predicting localized renal cell carcinoma recurrence when compared with a clinicopathological characteristics’ scoring system (SSIGN score).

We acknowledge that our study has limitations: This was a retrospective trial. The population was small and characterized by two predefined distinct groups of patients selected a priori on recurrence-free survival of <1 years vs. >5 years.

However, our genomic signature enhances risk stratification. Future prospective studies, with large cohorts of patients, will be necessary to validate this signature.

## 5. Conclusions

This genomic panel could drive treatment personalization and support different surveillance programs to improve management of two distinct ccRCC prognostic groups.

## Figures and Tables

**Table 1:** Patients’ characteristics (valid cases and percentages).

	RFS > 5 Years (N = 24)	RFS < 1 Year (N = 22)	*p* Value
**Sex**			
**Male**	13 (54.2)	11 (50.0)	0.77
**Female**	11 (45.8)	11 (50.0)	
**Age (Median, range)**	52 (34–76)	65 (49–82)	0.001
**Grade**			
**G1**	4 (17.4)	2 (9.1)	0.55
**G2**	8 (34.8)	6 (27.3)	
**G3**	11 (47.8)	13 (59.1)	
**G4**	0	1 (4.5)	
**Necrosis**			
**Yes**	7 (29.2)	8 (40.0)	
**No**	17 (70.8)	12 (60.0)	0.45
**ukn**	0	2	
**Sarcomatoid component**			
**Yes**	0	2 (9.5)	
**No**	24 (100)	19 (90.5)	0.12
**unknown**	0	1	
**Stage**			
**I**	15 (62.5)	6 (27.3)	
**II**	4 (16.7)	2 (9.1)	0.02
**III**	5 (20.8)	11 (63.6)	
**Karnofsky**			
**100**	23 (95.8)	6 (27.3)	
**80–90**	1 (4.2)	13 (59.1)	<0.0001
**60–70**	0	3 (13.6)	
**Surgery**	2 (8.3) 22 (91.7)		0.01
**Radical**		9 (40.9)	
**Partial nephrectomy**		13 (59.1)	

Abbreviations: Recurrence-free survival (RFS).

**Table 2:** List of differentially expressed genes between recurrence-free survival (RFS) of <1 y and RFS of >5 yrs.

Gene	Log2 Fold Change	Std Error (log2)	Lower Confidence Limit (log2)	Upper Confidence Limit (log2)	Linear Fold Change	Lower Confidence Limit (Linear)	Upper Confidence Limit (Linear)	*p*-Value	BONF. *p*.Value
IL6	−3.72	0.545	−4.79	−2.65	0.076	0.0363	0.159	2.91 × 10^−8^	4.98 × 10^−6^
G6PD	−1.16	0.21	−1.57	−0.75	0.447	0.336	0.595	1.98 × 10^−6^	3.39 × 10^−4^
TALDO1	−1.02	0.203	−1.42	−0.627	0.492	0.373	0.647	9.34 × 10^−6^	1.60 × 10^−3^
POLD4	−0.769	0.165	−1.09	−0.446	0.587	0.469	0.734	3.37 × 10^−5^	5.77 × 10^−3^
SQSTM1	−0.686	0.152	−0.984	−0.388	0.622	0.506	0.764	5.30 × 10^−5^	9.06 × 10^−3^
CP	−2.32	0.52	−3.34	−1.3	0.2	0.0986	0.405	6.13 × 10^−5^	1.05 × 10^−2^
DBN1	−1.21	0.273	−1.75	−0.677	0.432	0.298	0.626	6.73 × 10^−5^	1.15 × 10^−2^
TMEM8A	−0.768	0.176	−1.11	−0.423	0.587	0.463	0.746	8.24 × 10^−5^	1.41 × 10^−2^
TBC1D7	−0.735	0.173	−1.07	−0.395	0.601	0.475	0.76	1.24 × 10^−4^	2.12 × 10^−2^
SERPINA3	−4.03	0.97	−5.93	−2.13	0.0613	0.0164	0.229	1.62 × 10^−4^	2.78 × 10^−2^
GIPC1	−0.678	0.165	−1	−0.354	0.625	0.5	0.783	1.90 × 10^−4^	3.25 × 10^−2^
BAP1	−0.641	0.157	−0.949	−0.333	0.641	0.518	0.794	2.01 × 10^−4^	3.44 × 10^−2^
TKT	−0.645	0.158	−0.954	−0.335	0.64	0.516	0.793	2.02 × 10^−4^	3.45 × 10^−2^
TLCD1	−1.53	0.377	−2.27	−0.789	0.347	0.208	0.579	2.21 × 10^−4^	3.77 × 10^−2^
SLC4A3	−2.3	0.567	−3.41	−1.18	0.204	0.0944	0.44	2.21 × 10^−4^	3.78 × 10^−2^
PKM	−0.674	0.168	−1	−0.344	0.627	0.498	0.788	2.55 × 10^−4^	4.36 × 10^−2^
MTX1	−0.67	0.169	−1	−0.339	0.628	0.5	0.791	2.85 × 10^−4^	4.88 × 10^−2^

**Table 3:** Cox proportional hazard model with overall survival as outcome.

	UNIVARIATE HR (95% CI)	MULTIVARIATE HR (95% CI)
GENDER		--
(male vs. female)	0.95 (0.41–2.19) *p* = 0.90	
AGE		--
(>60 vs. <60 years)	2.06 (0.86–4.93) *p* = 0.11	
KARNOFSKY PS		
(100 vs. <100%)	0.15 (0.06–0.37) *p* < 0.0001	0.14 (0.05–0.34) *p* < 0.0001
GRADE		--
(G3–G4 vs. G1–G2)	1.14 (0.46–2.79) *p* = 0.78	
NECROSIS		--
(yes vs. no)	1.90 (0.75–4.83) *p* = 0.75	
STAGE		--
(III–IV vs. I–II)	2.64 (1.09–6.41) *p* = 0.03	
SURGERY		--
(radical vs. partial)	2.25 (0.90–5.62) *p* = 0.08	
SSIGN		
(for each point)	1.23 (1.05–1.44) *p* = 0.003	1.28 (1.07–1.52) *p* = 0.006
SIGNATURE		
(UGS vs. FGS)	51.37 (6.75–390.96) *p* < 0.0001	Not considered

Abbreviations: hazard ratio (HR); unfavorable geneomic signature (UGS); favorable genomic signature (FGS).

## Data Availability

The data are reported on medical records and available on request.

## Supplementary Materials

The following supporting information can be downloaded at: https://www.mdpi.com/article/10.3390/cancers14010178/s1. Table S1: list of all genes evaluated.

## Abbreviations

Polybromo 1 (PBRM1) BRCA-associated protein-1 (BAP1, 6–15%), SET domain containing 2 (SETD2, 3–11%), tumor protein 53 (TP53, 5%), lysine (K)-specific demethylase 5C (KDM5C, 3–5%), phosphatidylinositol-4,5-bisphosphate 3-kinase catalytic subunit alpha (PIK3CA, 3%), ataxia telangiectasia mutated (ATM, 3%), tuberous sclerosis complex subunit 1 (TSC1, 3%), AT-rich interaction domain 1A (ARID1A, 2%), Cyclin-dependent kinase inhibitor 2A (CDKN2A, 2%), vascular endothelial growth factor (VEGF), tyrosine kinase inhibitors (TKIs).
